# Effects of multiple factors on particle size selectivity under artificial extreme rainfall events on simulated Gobi surface

**DOI:** 10.1038/s41598-023-50136-x

**Published:** 2023-12-27

**Authors:** Liying Sun, Qingyuan Dai, Ziheng Feng

**Affiliations:** 1 Key Laboratory of Water Cycle and Related Land Surface Processes, Institute of Geographic Sciences and Natural Resources Research, Chinese Academy of Sciences, Beijing, 100101 China; 2 College of Resources and Environment, University of Chinese Academy of Sciences, Beijing, 100049 China

**Keywords:** Hydrology, Environmental impact

## Abstract

Understanding multiple-factor effects on particle size selectivity by extreme rainfall events in Ala-Shan Gobi desert is of great significance for better estimation of potential Asian dust emission sources. Artificial rainfall simulation experiments were used to investigate the particle size selectivity characteristics by extreme rainfall events under different rainfall intensities (20 mm h^−1^ and 40 mm h^−1^), slope gradients (3° and 15°) and gravel coverages (0, 30%, and 60%). Moreover, the relations of clay content (*Clc*), silt content (*Sic*), fine particle (< 50 μm) content (*Fic*) and enrichment ratio of fine particles (*ER*_<50_) with multiple factors were regressed and validated. Results show that rainfall intensity significantly (*P* < 0.05) affect runoff and sediment yield processes, but slope gradient was a dominant factor that changed particle size distribution (PSD). The selectivity of fine particles was higher at low rainfall intensity (20 mm h^−1^), gentle slope (3°) and moderate gravel coverage (30%), with *ER*_<*50*_ reaching 6.14, which dominate the potential Asian dust emission sources. The interaction were discussed and classified into ‘Synergy’ and ‘Trade-off’. *Clc* and *Fic* showed negative exponential relationship with rainfall intensity and slope gradient, but positive exponential relationship with gravel coverage. While *Sic* and *ER*_<*50*_ showed negative power function relationship with rainfall intensity, slope gradient and gravel coverage. These findings could help to understand the effects of multiple factors on potential sources of Asian dust emission under extreme rainfall events in Gobi region of northwestern China and provide basic science reference for the prediction of dust emission in this region.

## Introduction

In China, Gobi desert or ‘desert with a gravel surface’ is mainly located in the northwestern arid region, with an area of 72 × 10^4^ km^2^^1,2^. Frequent occurring of sand or dust storms by wind erosion in northwestern China damaged cultivated lands, constructions and traffic routes^2^. Long-range transportation of suspended dust storms by westerly winds becomes a part of the global dust cycle, and has effect on biogeochemical cycles, solar radiation balance and even sea-land materials in Northeastern Asia and distant North Pacific^1,3–10^. Gobi deserts of China are recognized as major potential sources of dust emission in Central Asia^4,11,12^.

Previous investigations demonstrated that Gobi surface has strong resistance to wind erosion, and only erodible fine particles (< 50 µm) could be possible sources of dust emission in Ala-Shan Gobi desert^2,12^. Despite existing inconsistent opinions, particles sorted by rainfall or ephemeral streams are recognized as an important potential source of these erodible fine particles^12–15^. For example, Wang et al. indicated that one important source of fine particles in Ala Shan Gobi desert is from water erosion processes by ephemeral streams. We also found water erosion by rainfall in Ala Shan Gobi desert^12,16^.

Particle size selectivity is a natural phenomenon during erosion processes by rainfall and receives increasing concerns due to its complexity^17–19^. Different size fractions were found to be sorted by different mechanisms^20,21^. For example, finer particles (< 50 µm) with clay and silt composition are more likely to be eroded than coarser ones and transported through suspension^18,22–24^. In addition, coarser particles may increase with rainfall duration and can be transported by bed-rolling^21,23^. In the context of global warming, it was reported that temperature in northwestern China has increased 0.36 °C per decade, which is approximately triple of the global average^25,26^. Accordingly, precipitation in northwestern China showed an increasing trend with spatial difference^27^. The frequency of the extreme precipitation also showed an increasing trend with the rising temperature^28^. The extreme rainfall event, with significant higher rainfall intensity than the history recorded, may occurs in the future with higher frequency under climate change. Thus, it is necessary to understand the particle size selectivity processes under the extreme rainfall events and their further effects on the potential sources of dust emission to provide scientific reference for better projection of dust emission in the Gobi region in the future under high frequency of extreme rainfall events.

Besides important influences of rainfall characteristics, other factors also impact size selectivity processes, like original soil properties^29,30^, slope gradient^29,31^, soil surface coverage^17,32^ and antecedent soil moisture^22,33^. Previous investigations indicated that the composition of the eroded sediment would be similar to original soils in sufficient erosion conditions^23,34^. Fractions of silts were observed to show decreasing tendency with the increase of slope gradient during rainfall experiments by Han et al.^22^. However, an enrichment ratio of clay and silt fractions showed no significant differences with variations of slope gradient in study by Vaezi et al.^31^. The detachment of different sediment fractions is severely affected by soil surface cover through its influences on hydrodynamics of raindrop and overland flows^19^. Koiter et al.^29^ indicated interactions of slope gradients, vegetation cover and antecedent soil moisture contents on the enrichment of fine particles (< 63 µm) by interrill erosion.

Continuous pavement with gravel coverage is one of important surface properties in Gobi deserts, and gravels are ubiquitous in the Gobi desert with high spatial differences^2,12,35,36^. According to Zhang et al.^37^, the gravel coverage ranged at 22–91% in northwestern China. However, the influences of these gravels on particle size selectivity by extreme rainfall events in Gobi desert region were not well revealed, as well as the effects of other influencing factors like rainfall intensity and slope gradient.

In this study, laboratory artificial rainfall experiments were conducted with soils collected from the Ala Shan Gobi desert under extreme rainfall events with rainfall intensities (20 mm h^−1^ and 40 mm h^−1^) nearly 2 and 4 times of the history maximum rainfall intensity, two slope gradients (3° and 15°) and three gravel coverages (0, 30%, and 60%). Main objectives of this article are: (i) to analyze the effects of rainfall intensity, slope gradient and gravel coverage on the characteristics of particle size selectivity induced by extreme rainfall events; (ii) to understand interactions of multiple influencing factors on the particle size selectivity by extreme events. The study is of significance to understand the effects of multiple factors on potential sources of Asian dust emission under extreme rainfall events in Gobi region of northwestern China and provide basic scientific reference for the prediction of dust emission in this region.

## Materials and methods

### Experimental soils

**Figure 1:**
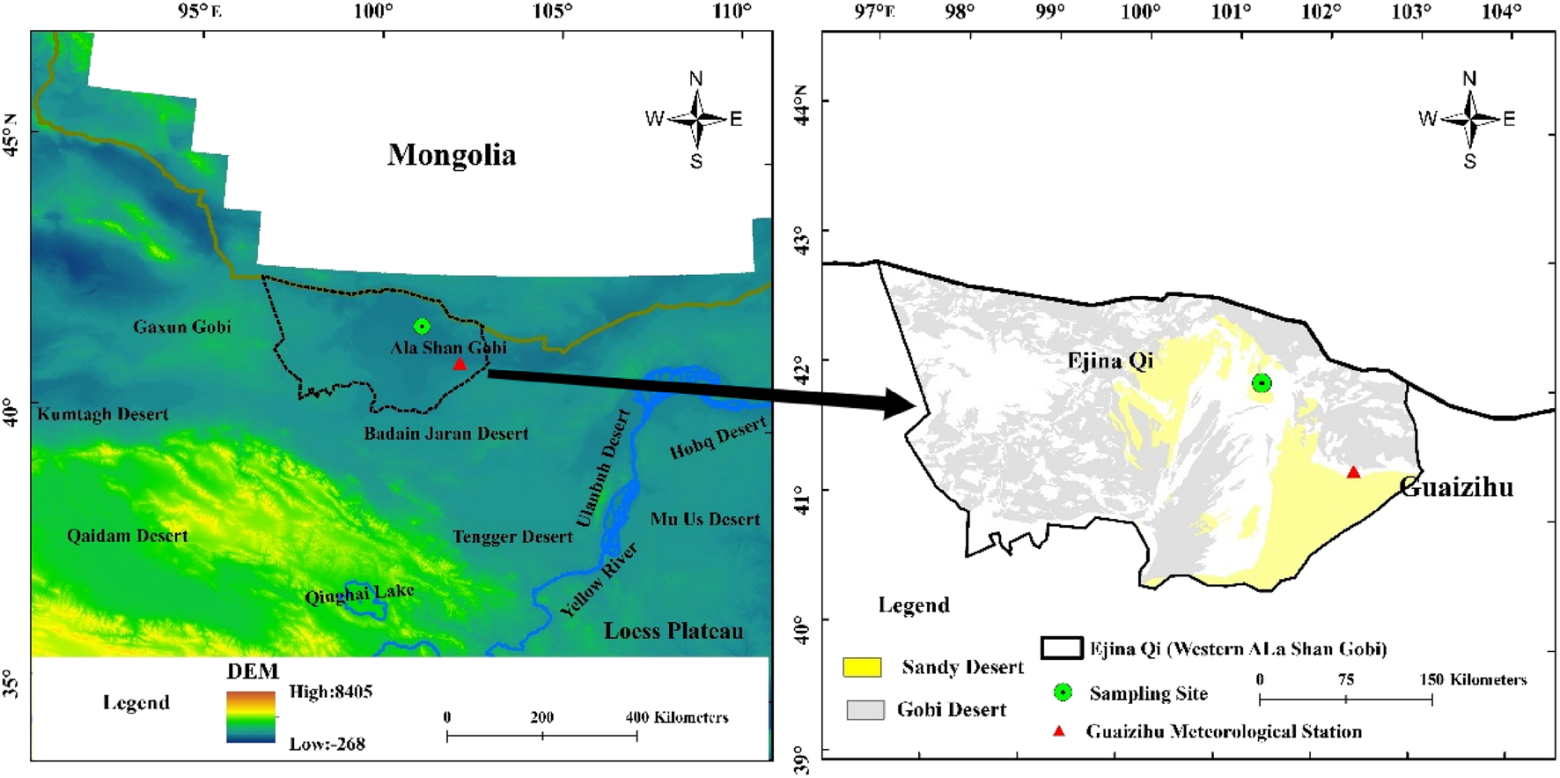
Location of soil sampling site in the Ala Shan Gobi desert and the slope gradient distribution in Ejina Qi County.

Experimental soils were collected from the western Ala-Shan Gobi desert of Inner Mongolia (42°01′ N, 101°22′ E) in China (Fig. 1). Samples were collected up to depth of 40 cm. Experimental soil samples were air dried naturally to soil content at 2.70% and passed through a 2-mm sifter for the separation of soils and stones. Then the stones were passed through a 10-mm sifter. The sieved soils (< 2 mm) and stones (2–10 mm) were prepared to simulate the Gobi surface for the artificial rainfall experiments. The tested clay (< 2 µm) content, silt (2–50 µm) content and sand (50–2000 μm) content in the sieved is 2.72%, 9.73% and 87.56%, respectively. Besides, the median particle diameter (*d*_*50*_) of the sieved soil is 190 µm.

### Experimental equipment

Movable steel boxes (2 m × 1 m × 0.4 m; length × width × height) and the artificial simulated rainfall system (EL-RS3/5) with Veejet 80,100 nozzels were used for laboratory simulation experiments at State Key Laboratory of Earth Surface Processes and Resource Ecology, Beijing China. Homogeneity of rainfall intensity was ensured at higher than 90%.

### Experimental conditions

According to the global precipitation data (GPM (IMERG V06)), the rainfall intensity ranged at 0–133.91 mm h^−1^ with the time resolution of 30 min in the northwestern region of China in the past ten years (2012–2022). The average annual rainfall amount (45 mm) and maximum rainfall intensity (9 mm h^−1^) were recorded from Guaizihu Meteorological Station (41°13′N, 102°22′E, 960 m a.s.L) nearest the sampling site. Considering the increasing trend of both rainfall intensity and the frequency of the extreme rainfall events in the context of climate change^27,28^, the experimental rainfall intensities were set as 20 mm h^−1^ and 40 mm h^−1^ (nearly 2 times and 4 times of the history maximum rainfall intensity at Guaizihu Meteorological Station) to simulate the extreme rainfall events in the field of the Ala Shan Gobi with the global warming. The rainfall duration was set at 60 min for all artificial experiments.

According to the extraction data of slope distribution of sampling region by ArcGIS (10.3), the steep slope gradient could reach 22°, despite 95.7% of the area with slope gradient less than 3° (Fig. 1). Thus, two slope gradients (3° and 15°) were set for the indoor simulation experiments.

**Figure 2:**
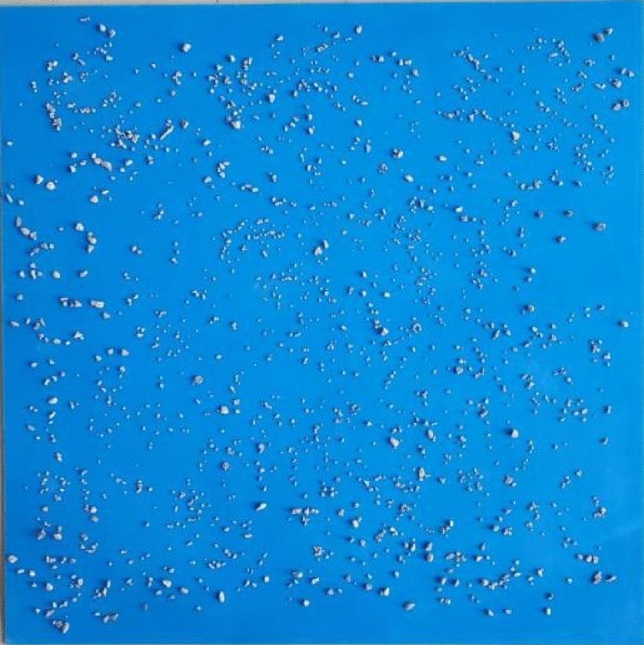
Experimental gravel distribution on an 1m × 1m blue PVC plastic plate.

The actual gravel coverage in sampling site was reported to range from 18% to 43% in photographic analyses, and 30% gravel coverage was a critical coverage in sediment transportation by wind in Ala Shan Gobi^12^. Hence, the gravel coverage was set at 0%, 30% and 60%, respectively. The setting of the experimental gravel coverage indoor is based on relation of gravel coverage and gravel mass. As shown in Fig. 2, the well mixed experimental gravels (2–10 mm) were weighed evenly spread on a 1m × 1m blue PVC plastic plate and photographed vertically with an automatic optical camera (about 3 m above). After that, the photos were processed and categorized by PHOTOSHOP software (2020) for the determination of gravel coverage on the surface. The relation of the gravel coverage and gravel mass was established by multiple sets (5 sets with more than 3 times shoot for each set) as shown in Eq. (1).

1 $$y = 10.55x - 0.038 R^{2} = 0.908$$

where, *y* is the gravel coverage (%) and *x* is the gravel mass (kg).

### Experimental processes and measurements

#### Filling experimental soils

Firstly, soils were filled into movable boxes with permeable gauze at the bottom of the box to ensure water infiltration from holes at the bottom of the box for water infiltration. The filling of experimental soils is from bottom to up by two layers with the soil bulk density controlling at 1.55 g cm^−3^ for the lower-layer (10–20 cm) and at 1.45 g cm^−3^ for the upper layer (0–10 cm). Then, the prepared stones (2–10 mm) were weighed according to the relation of gravel coverage and gravel mass for the simulation of different gravel coverage (0%, 30% and 60%) and spread evenly on the surface of the filled slope.

#### Experimental measurements

The whole experimental processes were recorded with video cameras for the determination of the erosion processes. No rills were observed for all experiments. Runoff were measured by 16-L plastic buckets. Besides, 500-mL plastic bottles were used to collect runoff samples at 3-min intervals for sediment concentration measurement by the method of oven-drying (105 °C). A 100-mL glass baker was used to collect the runoff for the determination of the particle size distribution (PSD) in the eroded sediment by the Malvern Mastersizer 2000 laser diffraction device (Malvern Instruments Ltd.). All experiments were conducted with duplicate tests.

### Data analysis

#### Particle size selectivity characterization

Three indicators were applied to characterize the particle size selectivity during the erosion process: (i) PSD; (ii) the median particle diameter (*d*_*50*_); and (iii) the enrichment ratio of fine particles (*ER*_<*50*_). PSD is further classified into three grades, *i.e.*, clay fraction (< 2 µm), silt fraction (2–50 µm) and sand fraction (50–2000 µm). The smaller *d*_*50*_ is in the eroded sediment, the finer sediment particles are eroded or transported^32^. *ER*_<*50*_ is used to indicate the selective of clay and silt fractions during the erosion processes, which is calculated as the ratio of fine particles (< 50 µm) content in the eroded sediment to fine particles (< 50 µm) content in the original soil^22^.

#### Statistic analysis

The differences of PSD, *d*_*50*_ and *ER*_<*50*_ in the eroded sediment under different experimental conditions were analyzed by Analysis of Variance (ANOVA) with the least significant difference (LSD) procedure at 95% confidence (SPSS 26.0). Differences analysis of variables between two rainfall intensities and two slope gradients were analyzed by the paired *T*-test. Multiple regression analysis (SPSS 26.0) was applied for the determination of the relations between variables of particle size selectivity and multiple influencing factors. Furthermore, regression equations were validated by the coefficient of determination (*R*^2^) and the Nash coefficient (*E*_*NS*_) with a set of independent data from duplicate experiments.

#### Interaction effects analysis

Multi-way analysis of variance by SPSS (26.0) was applied to determine whether the interactions of multiple factors have significant effects on the observed soil particle size selectivity variables at 95% confidence with *P* < 0.05^38^. The interactions of pairwise factors included rainfall intensity and slope gradient (*RI*-*SG*), rainfall intensity and gravel coverage (*RI*-*GC*) and slope gradient and gravel coverage (*SG*-*GC*). Then the type of the interaction effects by pairwise factors were classified into synergy effects and trade-off effects based on the contribution by each single factor to the changes of variables (PSD, *d*_*50*_ and *ER*_<*50*_). Synergy effects mean that the single factor of pairwise factors has identical contribution to variables (PSD, *d*_*50*_ and *ER*_<*50*_), while trade-off effects mean that the single factor of pairwise factors has the opposite contribution to variables (PSD, *d*_*50*_ and *ER*_<*50*_).

## Experimental results

### Runoff and sediment yield under different experimental conditions

#### Runoff yield

**Figure 3:**
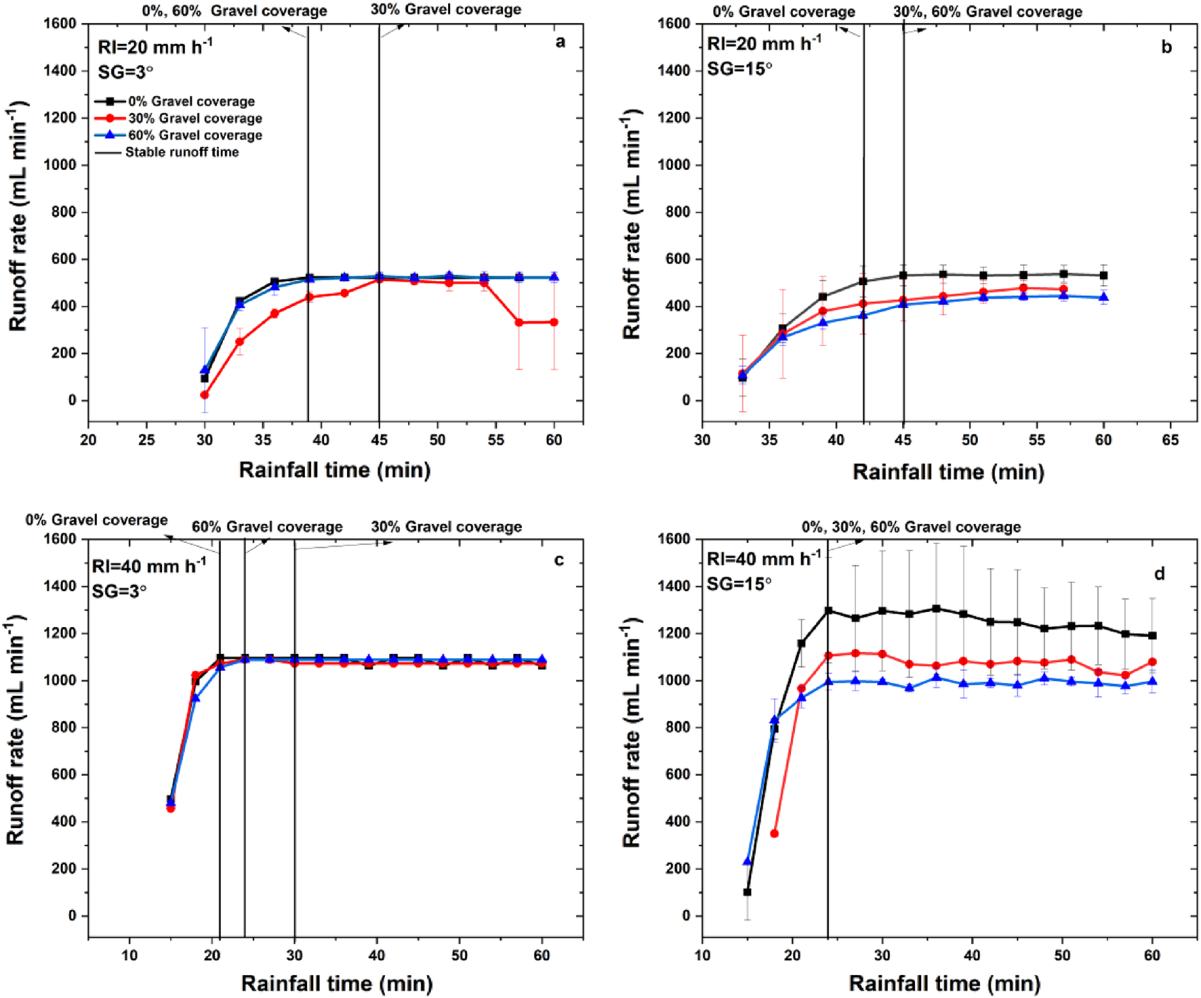
Runoff rate changes with rainfall duration under different experimental conditions (*RI* is rainfall intensity, *SL* is slope gradient, and *GC* is gravel coverage).

The runoff rate first increased with rainfall duration and then kept stable under different experimental conditions (Fig. 3), and this phenomenon is consistent with previous studies in the semi-arid region of northwestern China^39^. Stable runoff time (*SR*_*time*_) was determined when the changing rate of the runoff was less than 5%. As shown in Table 1, runoff yield time (*RT*) ranged at 15.5–31.5 min and the mean runoff rate after stable (*SR*_*mean*_) ranged at 483.9–1087.9 mL min^−1^ under different experimental conditions. Both *RT* and *SR*_*time*_ showed significant (*P* < 0.05) decreasing trend but *SR*_*mean*_ showed significant (*P* < 0.05) increasing trend with the increase of rainfall intensity. However, these variables did not show significant differences with changes of slope gradient and gravel coverage.

**Table 1:** Runoff and sediment characteristics under different experimental conditions.

Experiment conditions		*SR*_*mean*_ (mL min^−1^)	*SL*_*mean*_ (g min^−1^)	*RT* (min)	*SR*_*time*_ (min)
Rainfall Intensity (mm h^−1^)	20	483.9 ± 45.3^a^	38.8 ± 16.3^a^	32.5 ± 2.3^a^	42.5 ± 3.0^a^
	40	1087.9 ± 88.6^b^	62.1 ± 16.3^b^	15.5 ± 1.2^b^	23.5 ± 1.2^b^
Slope gradient (°)	3	782.6 ± 313.8^a^	48.7 ± 19.6^a^	23.0 ± 8.8^a^	32.0 ± 10.2^a^
	15	789.2 ± 361.0^a^	52.3 ± 21.5^a^	25.0 ± 10.0^a^	34.0 ± 11.0^a^
Gravel coverage (%)	0	837.2 ± 367.0^a^	52.4 ± 22.6^a^	24.0 ± 10.4^a^	31.5 ± 10.5^a^
	30	764.6 ± 364.9^a^	52.2 ± 22.7^a^	24.8 ± 9.9^a^	34.5 ± 12.1^a^
	60	755.8 ± 325.5^a^	46.9 ± 19.3^a^	23.3 ± 9.6^a^	33.0 ± 10.7^a^

Different lowercase letters represent significant (*P* < 0.05) differences among variables under different experimental conditions; *SR*_*mean*_ is the mean runoff rate after stable runoff; *SL*_*mean*_ is the mean sediment load after stable runoff; *RT* is the runoff yield time; *SR*_*time*_ is the stable runoff time.

#### Sediment yield

**Figure 4:**
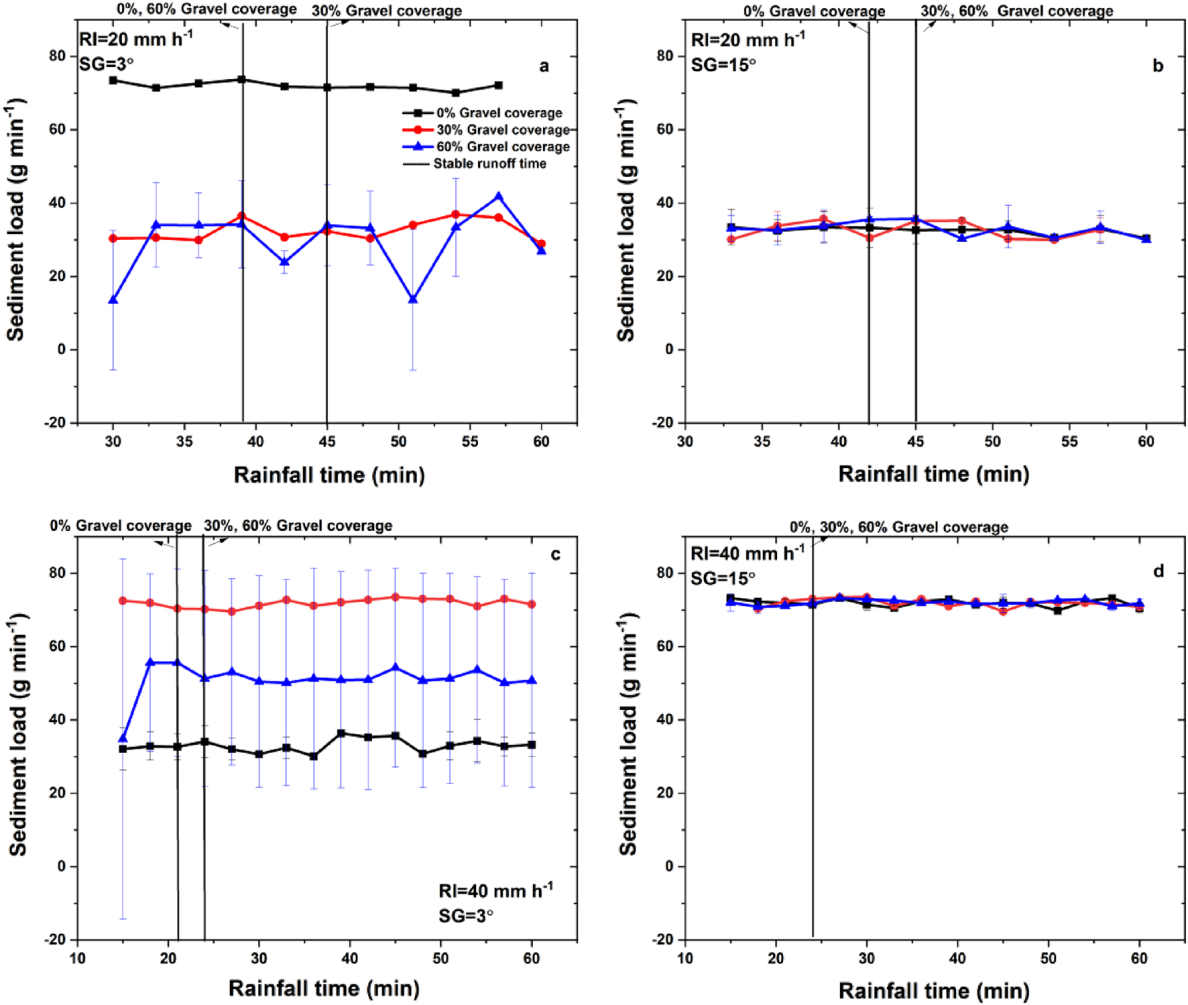
Sediment yield changes with rainfall duration under different experimental conditions.

As shown in Fig. 4, sediment load showed higher variations on 3° slope than those on 15° slope. The mean sediment load (*SL*_*mean*_) ranged at 38.8–62.1 g min^−1^, showing significant (*P* < 0.05) increasing trend with the increase of rainfall intensity and no significant (*P* < 0.05) differences with changes of slope gradient and gravel coverage.

### Changes of PSD in the eroded sediment under different experimental conditions

**Figure 5:**
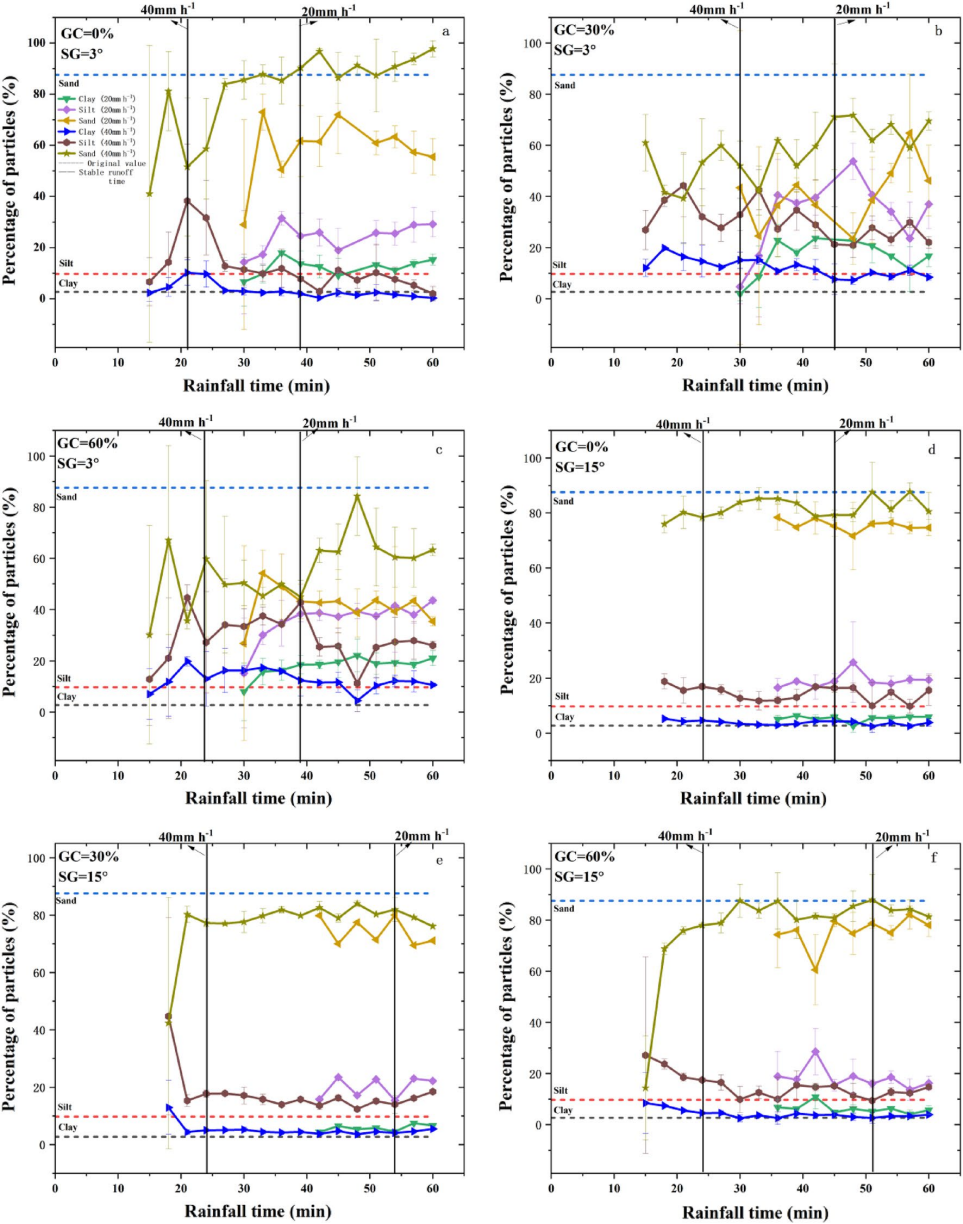
Changes of PSD in the eroded sediment with rainfall duration under different experimental conditions.

As shown in Fig. 5, PSD in the eroded sediment varied with the experimental conditions. The contents of clay, silt and sand in the eroded sediment ranged at 2.1%–22.3%, 2.0%–43.2% and 30.0%–95.0%, respectively, under different experimental conditions. The changes of different size fractions with rainfall duration showed higher fluctuations at gentle slopes (3°; Fig. 5a–c), while kept stable at steeper slope (15°; Fig. 5d–f).

At gentle slopes (3°), clay and silt contents showed increasing trends with rainfall duration under 20 mm h^−1^, whilst they first increased and then decreased with rainfall duration under 40 mm h^−1^. However, both rainfall intensity and gravel coverage did not change the increasing trend of sand contents with rainfall duration at gentle slopes (3°).

As shown in Table 2, silt content (*Sic*) after rainfall was significantly (*P* < 0.05) higher than the original value under different experimental conditions, the clay content (*Clc*) after rainfall was significantly (*P* < 0.05) higher than the original value except experimental conditions at 15° slope or at 0% coverage and the fine particle (< 50 µm) content (*Fic*) after rainfall was significantly (*P* < 0.05) higher than the original value except experimental conditions at 0% coverage. The sand content (*Sac*) after rainfall was significantly (*P* < 0.05) lower than the original value under different experimental conditions.

**Table 2:** Differences of PSD, *d*_*50*_ and *ER*_<*50*_ in the eroded sediment under experimental conditions.

Experiment Conditions		PSD				*d*_*50*_ (μm)	*d*_*50peak*_ (μm)	*ER*_<*50*_
		Clay content/*Clc* (< 2 μm/%)	Silt content/*Sic* (2–50 μm/%)	Sand content/*Sac* (50–2000 μm/%)	Fine Particle content/*Fic* (< 50 μm/%)			
Origin value		2.7 ± 0.7^A^	9.7 ± 2.7^A^	87.6 ± 3.4^A^	12.4 ± 3.4^A^	194.7 ± 34.3^A^	–	–
Rainfall Intensity (mm h^−1^)	20	11.3 ± 6.4^Ba^	26.2 ± 8.9^Ba^	62.5 ± 15.2^Ba^	37.5 ± 15.2^Ba^	130.3 ± 49.2^Ba^	430.8 ± 165.0^a^	3.0 ± 1.2^a^
	40	6.9 ± 4.4^Ba^	19.9 ± 7.8^Ba^	73.1 ± 12.2^Ba^	26.9 ± 12.2^Ba^	232.7 ± 83.9^Ab^	573.4 ± 105.1^a^	2.2 ± 1.0^a^
Slope Gradient (°)	3	13.1 ± 5.7^Ba^	28.6 ± 9.2^Ba^	58.3 ± 14.8^Ba^	41.8 ± 14.8^Ba^	179.4 ± 118.9^Aa^	580.3 ± 136.5^a^	3.3 ± 1.2^a^
	15	5.1 ± 0.9^Ab^	17.5 ± 2.1^Bb^	77.4 ± 3.0^Bb^	22.6 ± 3.0^Bb^	183.6 ± 40.2^Aa^	504.1 ± 63.2^a^	1.8 ± 0.2^b^
Gravel Coverage (%)	0	6.3 ± 4.5^Aa^	17.9 ± 5.8^Ba^	75.8 ± 10.1^Aa^	24.2 ± 10.1^Aa^	221.6 ± 114.7^Aa^	593.4 ± 82.8^a^	1.9 ± 0.8^a^
	30	10.4 ± 6.2^Ba^	26.2 ± 9.0^Ba^	63.5 ± 15.2^Ba^	36.6 ± 15.2^Ba^	151.7 ± 15.5^Aa^	447.6 ± 120.2^a^	2.9 ± 1.2^a^
	60	10.6 ± 6.6^Ba^	25.2 ± 10.2^Ba^	64.2 ± 16.8^Ba^	35.8 ± 16.8^Ba^	171.2 ± 96.9^Aa^	572.1 ± 129.3^a^	2.9 ± 1.3^a^

PSD is the particle size distribution; *d*_*50*_ is the median particle diameter; *d*_*50peak*_ is the peak value of the median particle; *ER*_<*50*_ is the enrichment ratio of fine.

Particles (< 50 µm);—represents no value; different capital letters show significant differences of variables (PSD, *d*_*50*_, *d*_*50peak*_) before and after rainfall events at *P* < 0.05; different lowercase letters show significant differences of variables (PSD, *d*_*50*_, *d*_*50peak*_, *ER*_<*50*_) under different experimental conditions at *P* < 0.05.

### Changes of ***d***_***50***_ in the eroded sediment under different experimental conditions

**Figure 6:**
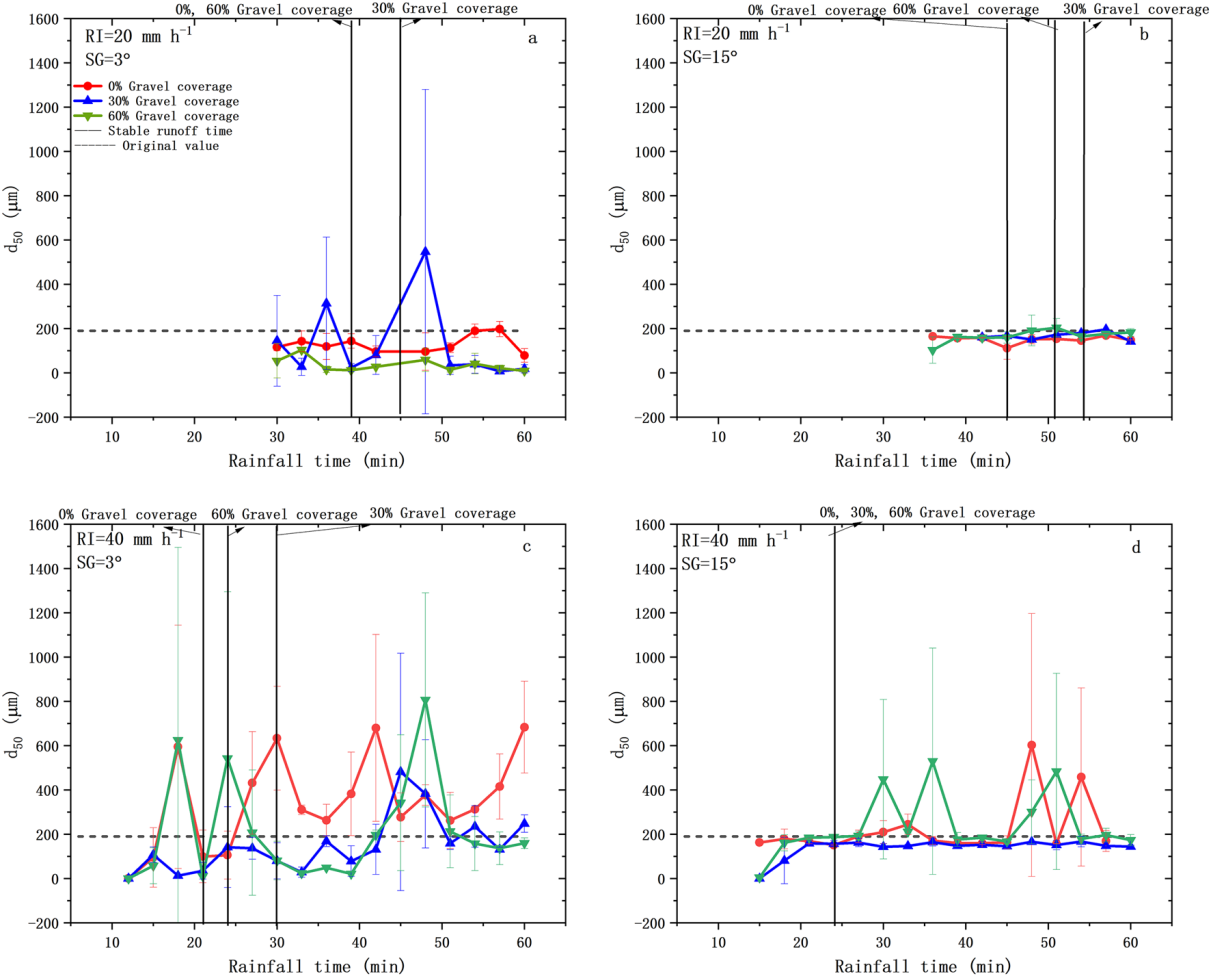
Changes of *d*_*50*_ in the eroded sediment under different experimental conditions.

As shown in Fig. 6, mean *d*_*50*_ ranged at 7.61–806.09 μm and the mean peak value of *d*_*50*_ (*d*_*50peak*_) ranged at 314.15–806.06 μm under different experimental conditions. The *d*_*50peak*_ only occurred at 30% gravel coverage under rainfall intensity of 20 mm h^−1^, whilst the *d*_50_ showed great fluctuations and occurred peak values except steep slope (15°) and moderate gravel coverage (30%) under rainfall intensity of 40 mm h^−1^. Compared with the original value, *d*_50_ in the eroded sediment after rainfall event was significantly (*P* < 0.05) lower under 20 mm h^−1^, but did not show significant (*P* < 0.05) differences under 40 mm h^−1^. In terms of the effects of different influencing factors, *d*_50_ showed significant (*P* < 0.05) increasing trend with the increase of rainfall intensity, but did not show significant (*P* < *0.05*) differences with variations of slope gradient and gravel coverage.

### ***ER***_<***50***_ under different experimental conditions

**Figure 7:**
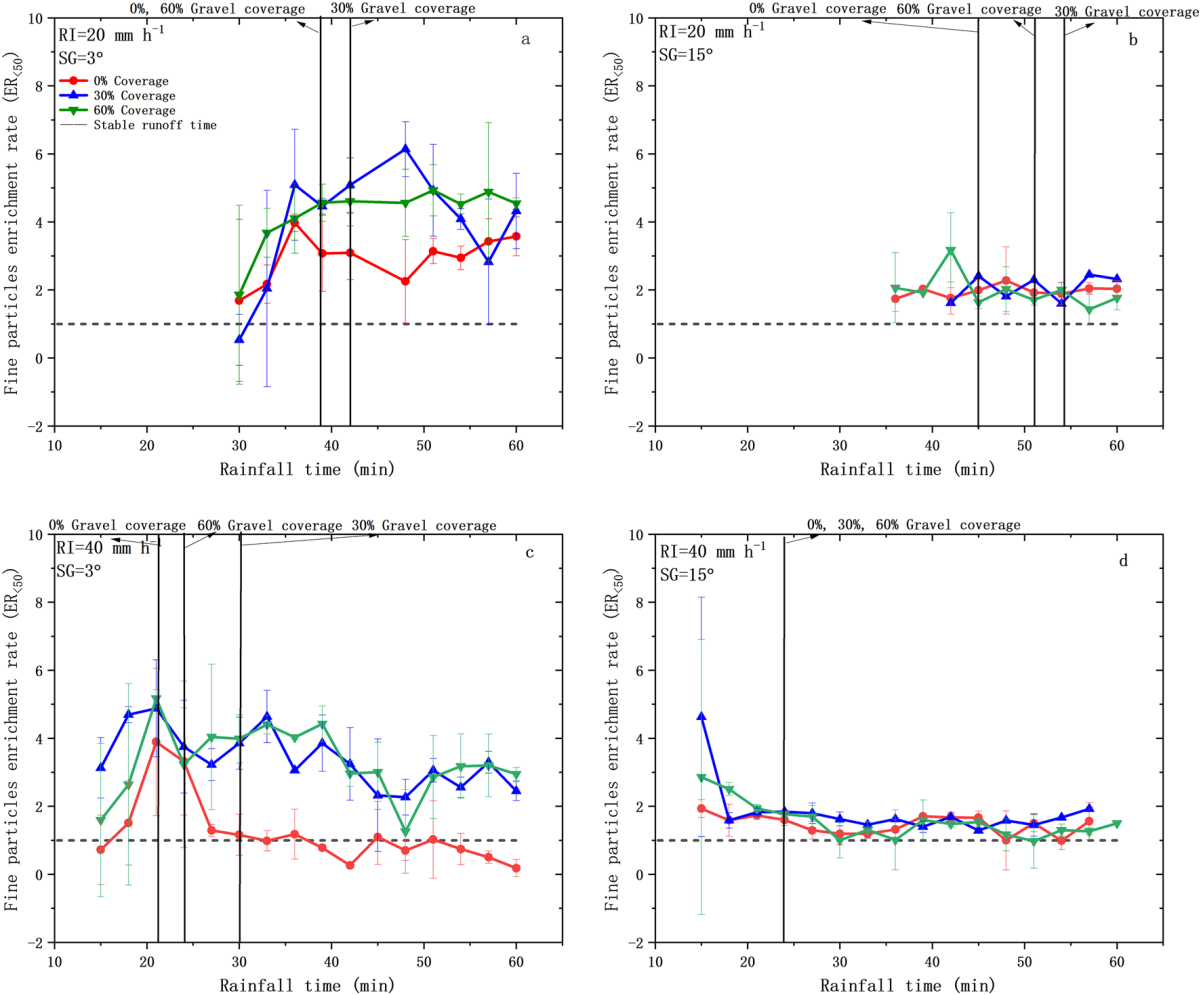
Changes of *ER*_<50_ in the eroded sediment under different experimental conditions.

As shown in Fig. 7, *ER*_<*50*_ showed higher fluctuations (more than 50%) at gentle slopes (3°; Fig. 7a,c) than that at steeper slopes (15°; Fig. 7b,d). At gentle slopes (3°), *ER*_<*50*_ showed dramatic variations with rainfall duration and gravel coverage, with the At 3° slopes, *ER*_<*50*_ showed increasing trend with rainfall duration under rainfall intensity of 20 mm h^−1^, whilst *ER*_<*50*_ first increased and then decreased with rainfall duration under rainfall intensity of 40 mm h^−1.^ At steep slopes (15°), *ER*_<*50*_ showed decreasing trend with rainfall duration under 40 mm h^−1^. At gentle slopes (3°), *ER*_<50_ ranged at 0.53–6.14 under 20 mm h^−1^ and at 0.18–5.18 under 40 mm h^−1^. *ER*_<50_ showed significant (*P* < 0.05) decreasing trend with the increase of slope gradient, ranging at 1.43–3.17 under 20 mm h^−1^ and at 0.97–4.63 under 40 mm h^−1^, respectively. In most experimental conditions (except 20 mm h^−1^ and 3°, 0% gravel coverage), fine particles were enriched in the eroded sediment with *ER*_<50_ > 1 (Table 2). Specifically, the selectivity of fine particles was higher at low rainfall intensity (20 mm h^−1^), gentle slope (3°) and moderate gravel coverage (30%), with *ER*_<*50*_ reaching 6.14. *ER*_<*50*_ did not show significant (*P* < *0.05*) differences with the variations of rainfall intensity and gravel coverage.

## Discussion

### Response of particle size selectivity to multiple factors

#### Rainfall intensity

Rainfall intensity played significant roles in runoff and sediment yield, as the mean runoff rate after stable (*SR*_*mean*_) and the mean sediment load after stable (*SL*_*mean*_) showed significant (*P* < 0.05) differences with rainfall intensity, but did not show significant differences with slope gradient and gravel coverage (Table 1). Clay content (*Clc*), silt content (*Sic*) and fine particle (< 50 µm; *Fic*) content showed significant (*P* < 0.05) differences before and after rainfall events under different experiments (Table 2), which is consistent with results of previous studies^18,22–24^.

Although variables of *Clc*, *Sic*, *Fic* and *ER*_<*50*_ showed decreasing trend with the increase of rainfall intensity, these variables did not show significant (*P* < 0.05) differences with rainfall intensity. The sand content (*Sac*) did not show significant differences with the rising rainfall intensity, however, *d*_50_ showed significant (*P* < 0.05) increasing trend when rainfall intensity increased from 20mm h^−1^ to 40 mm h^−1^. This is mainly due to the high sand content of the experimental soil.

#### Slope Gradient

As shown in Table 1 and Figs. 3 and 4, slope gradient did not show significant (*P* < 0.05) impacts on runoff and sediment yields, as *RT, SR*_*mean*_ and *SL*_*mean*_ did not show significant (*P* < 0.05) differences at different slope gradients. This is different with the results of higher runoff and sediment yield at steeper slopes in previous studies in other areas^19^. As shown in Table 2, *Clc*, *Slc*, *Fic* and *ER*_<*50*_ were significantly (*P* < 0.05) lower but *Sac* was significantly (*P* < 0.05) higher when slope gradient increased from 3° to 15°, which suggested that slope gradient was a dominant factor that significantly (*P* < 0.05) changed PSD. Fine particles are carried out in the runoff at gentle slope and coarse particles and small aggregates are transported with higher runoff kinetic energy at steeper slope gradients and thus increases *d*_*50*_ on 15° slope..However, the content of fine particles is weaken with the aggregating processes at steeper slopes^40^. Therefore, higher fine particle selectivity is shown at gentle slope (3°) with lower runoff energy, which is consistent with the previous study^41^.

#### Gravel coverage

Compared with bared surface (0% gravel coverage), fine particles (*Clc*, *Slc*, *Fic*) and *ER*_<50_ showed insignificant increasing trend under gravel coverage (30% and 60%). This suggested the insignificant increasing effects of gravel coverage on fine particle selectivity. *Slc* and *Fic* showed highest values and *d*_*50*_, *d*_50peak_ showed lowest values under gravel coverage of 30%, which suggested that 30% gravel coverage setting was the most important for sorting and distribution of fine particles in runoff. Rainfall infiltration increases with the increase of gravel coverage, and runoff rates decrease with increasing gravel coverage by dissipating water flow^42,43^, which further affects the surface runoff and fine particle selectivity^44,45^. Thus, higher gravel coverage within a certain range can affect fine particle selectivity more significantly.

### Interaction effects of multiple factors on particle size selectivity

Pairwise factors of rainfall intensity and slope gradient (*RI*-*SG*) and pairwise factors of slope gradient and gravel coverage (*SG*-*GC*) showed significant interaction effects on PSD, *d*_*50*_ and *ER*_<*50*_, but pairwise factors of rainfall intensity and gravel coverage (*RI*-*GC*) did not show significant interaction effects on PSD, *d*_*50*_ and *ER*_<*50*_ (Table 3).

**Table 3:** Interaction effects of multiple factors on PSD, *d*_*50*_ and *ER*_<*50*_*.*

Item	Clay content (*Clc*) (< 2 μm/%)		Silt content (*Sic*) (2–50 μm/%)		Sand content (*Sac*) (50–2000 μm/%)		Fine particle content (*Fic*) (< 50 μm/%)		*d*_50_		*ER*_<*50*_	
	Interaction effects	Trend	Interaction effects	Trend	Interaction effects	Trend	Interaction effects	Trend	Interaction effects	Trend	Interaction effects	Trend
*RI-SG*	synergy	decline	–	–	synergy	rise	synergy	decline	synergy	rise	synergy	decline
*SG-GC*	trade-off	decline	trade-off	decline	trade-off	rise	trade-off	decline	–	–	trade-off	decline

*PSD* is particle size distribution; *RI* is rainfall intensity; *SG* is slope gradient; *GC* is gravel coverage.

Specifically, the increase of both rainfall intensity and slope gradient could decrease *Clc*, *Sic*, *Fic* and *ER*_<*50*_ and increase *Sac* and *d*_*50*_. The synergy interaction effects of *RI*-S*G* resulted in the decline of *Clc*, *Fic* and *ER*_<*50*_, and led to the rise of *Sac* and *d*_*50*_. Steeper slopes and higher rainfall intensities increased runoff kinetic energy and resulted in the less fine particle selectivity, which is consistent with previous studies^18,29,40,46^.

Whilst, the increase of slope gradient decreased *Clc*, *Sic*, *Fic* and *ER*_<*50*_, however, these variables increased when gravel coverage increased from 0 to 30% and 60%. The trade-off interactions of *SG*-*GC* resulted in the decline of *Clc*, *Sic*, *Fic* and *ER*_<*50*_. In contrast, the increase of slope gradient increased *Sac*, but *Sac* decreased when gravel coverage increased from 0 to 30% and 60%. The trade-off interaction effects of *SG*-*GC* led to the rise of *Sac*. These results also suggested the dominant roles of slope gradient on particle size selectivity by rainfall.

### The relationship between fine particles selectivity and influencing factors

The relations between fine particles selectivity, including clay content (*Clc*), silt content (Sic), fine particle (< 50 μm) content (*Fic*) and *ER*_<*50*_ and influencing factors were regressed as Eqs. (2–5).

2 $$Clc = {28}.{\text{52e}}^{{ - 0.0{24}RI - 0.0{9}SG + 0.00{9}GC}} R^{{2}} = 0.{88}0,F = {54}.{86},n = {11}$$

3 $$Sic = {176}.{43}RI^{{ - 0.{257}}} \times SG^{{ - 0.{436}}} \times GC^{{ - 0.0{62} }} R^{{2}} = 0.{986},F = {126}0.{33},n = {1}0$$

4 $$Fic = {69}.{\text{27e}}^{{ - 0.0{16}RI - 0.0{61}SG + 0.00{8}GC}} R^{{2}} = 0.{792},F = {49}.{42},n = {11}$$

5 $$ER_{ < 50} = {26}.{1}0RI^{{ - 0.{359}}} \times SG^{{ - 0.{534}}} \times GC^{{ - 0.00{3}}} R^{{2}} = 0.{995},F = {2525}.{56},n = {11}$$

where, *RI* is rainfall intensity, *SG* is slope gradient, and *GC* is gravel coverage.

**Figure 8:**
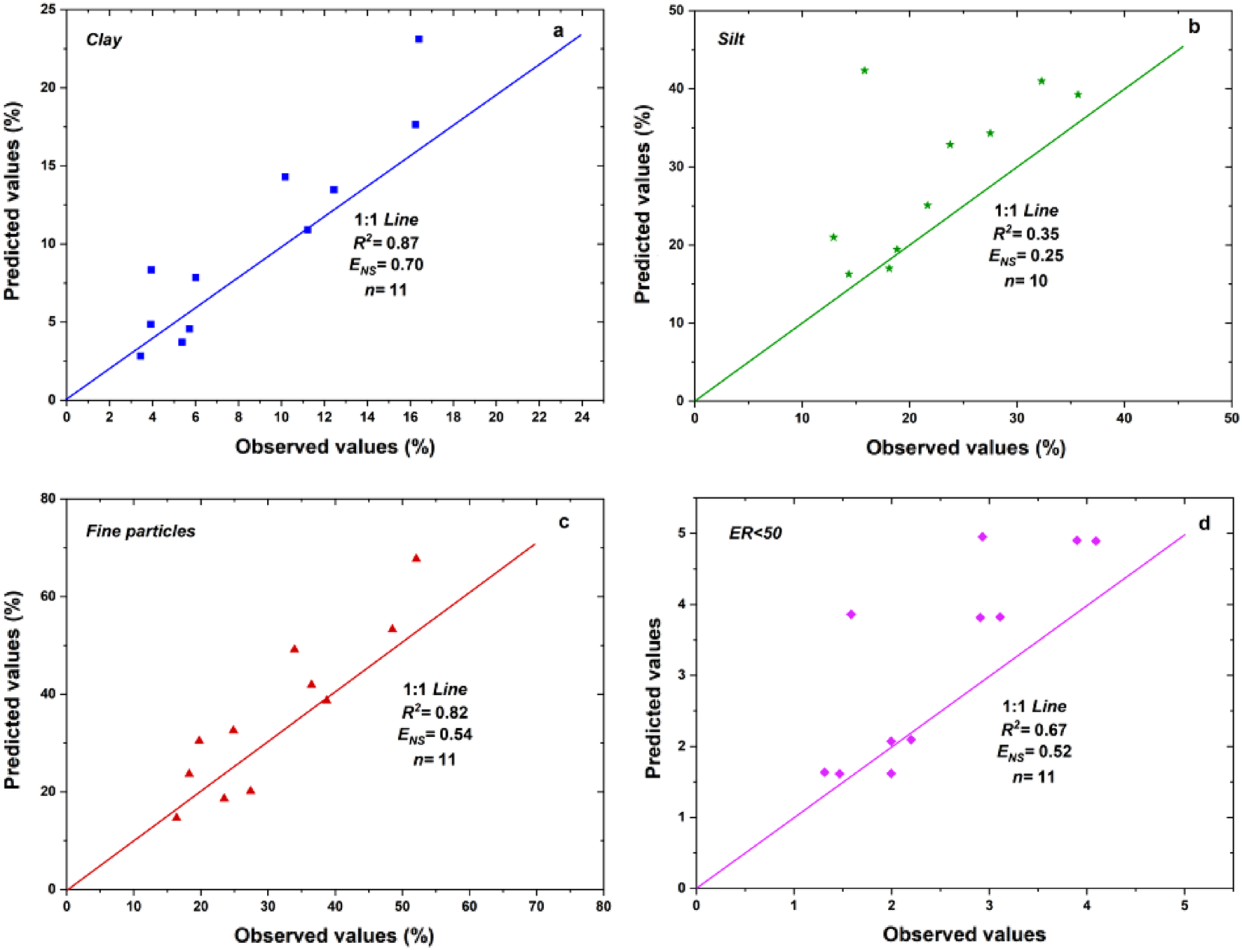
Comparison of predicted values and observed ones (*R*^2^ is a coefficient of determination and *E*_*NS*_ is a Nash coefficient).

Among them, *Clc*, *Fic* showed negative exponential relations with rainfall intensity and slope gradient and positive exponential relations with gravel coverage. *Sic* and *ER*_<*50*_ met negative power relations with rainfall intensity, slope gradient and gravel coverage. As shown in Fig. 8, the coefficient of determination (*R*^*2*^) and the Nash coefficient (*E*_*NS*_) were all > 0.5 for the regressed equations and this validated the accuracy of the prediction results with data of the duplicate experiments. Again, the slope gradient was demonstrated the dominant factor influencing the changes of PSD (*Clc*, *Sic* and *Fic*) and *ER*_<*50*_, as the coefficient of slope gradient was highest in Eqs. (2–5), followed by rainfall intensity and gravel coverage.

### Implications

In the Ala Shan Plateau, dust emissions appear to be controlled mainly by the availability of fine particles (< 50 µm) under relatively high wind velocity^12^. Thus, fine particles (< 50 µm) are recognized as the potential sources of dust emission. In our experiments, high selectivity of fine particles were shown as the fine particle (< 50 µm) after rainfall was significantly (*P* < 0.05) higher than the original value under gravel coverage. And the slope gradient was the most significant factor influencing the particle size selectivity with higher effects on potential sources of dust emissions (fine particle <  < 50 µm) on gentle slopes (< 3°) in the area of Ala Shan Gobi. Due to the high percent (approximately 95.7%) of gentle slopes (< 3°) in the Ala Shan Gobi, slope gradient effects could not be ignored when investigating dust emissions in the Gobi desert areas. Moreover, the synergy effects of pairwise factors rainfall intensity and slope gradient (*RI*-*SG*) and the trade-off effects of pairwise factors slope gradient and gravel coverage (*SG*-*GC*) could decline the potential sources of dust emission (fine particle <  < 50 µm), and the interaction effects of the pairwise factors (*RI*-*SG* and *SG*-*GC*) should also be well considering when predicting the dust emissions in the Gobi region.

## Conclusions

This study is of significance to investigate multiple factors impacts on particle size selectivity under extreme rainfall events using artificial rainfall experiments on the simulated Gobi surface. High selectivity of fine particles (< 50 μm) was shown under different experimental conditions, as clay content (*Clc*), silt content (*Sic*), fine particle (< 50 μm) content (*Fic*) after rainfall event were significantly (*P* < 0.05) higher than the original value before rainfall events. Among the multiple influencing factors, slope gradient is the dominant factor influencing particle size selectivity and has significant (*P* < 0.05) negative effects on the enrichment of fine particles (< 50 μm) in the eroded sediment by rainfall. Higher selectivity of fine particles (< 50 μm) is observed on lower rainfall intensity of 20 mm h^−1^ and 30% gravel coverage on gentle slope (3°). The significant (*P* < 0.05) synergy effects of *RI*-*SG* and the significant (*P* < 0.05) trade-off effects of *SG*-*GC* decline the selectivity of fine particles (< 50 μm) and reduce the enrichment of fine particles (< 50 μm) in the eroded sediment. The regression results showed the negative exponential relations between *Clc* and *Fic* with rainfall intensity and slope gradient and the negative power relations between *Sic* and *ER*_<*50*_ with rainfall intensity, slope gradient and gravel coverage. It is concluded that the potential sources of dust emission are most promoted on gentle slopes (≤ 3°) at 30% gravel coverage under rainfall intensity of 20 mm h^−1^ in the Ala Shan Gobi desert, as the fine particles (< 50 μm) were recognized as the potential sources of dust emission. The significant effects of slope gradient, the interactions of rainfall intensity and slope gradient (*RI*-*SG*) and the interactions of slope gradient and gravel coverage (SG-GC) should be well considered for the prediction of the dust emission in this region. Furthermore, field experiments should be carried out for better understanding the influencing effects of multiple factors on the potential sources of dust emission (fine particles < 50 μm) to make up for the limitations of the indoor experiments.

## Data Availability

The datasets used and/or analysed during the current study available from the corresponding author on reasonable request.
